# An analysis of children's clothing-related injuries cases reported by the media in mainland of China from 2003 to 2017

**DOI:** 10.1097/MD.0000000000019305

**Published:** 2020-02-28

**Authors:** Sixiang Cheng, Atipatsa Chiwanda Kaminga, Xunjie Cheng, Huilan Xu

**Affiliations:** a Department of Social Medicine and Health Management, Xiangya School of Public Health, Central South University,Changsha, Hunan Province; b College of Data Science and Information Engineering, Guizhou Minzu University, Guiyang, Guizhou Province; c Department of Epidemiology and Health statistics, Xiangya School of Public Health, Central South University, 238 Shangmayuanling Road ,Changsha, Hunan Province; d Department of Mathematics and Statistics, Mzuzu University, Private Bag 201, Luwinga, Mzuzu 2, Malawi.

**Keywords:** children's clothing-related injuries, epidemiological characteristics, media

## Abstract

**Background:**

Numerous reports in the media have suggested that children could be hurt due to their clothing, yet there are few medical reports that discussed children's clothing-related injuries. This study therefore, aimed to describe the epidemiological characteristics of media-reported injury cases related to children's clothing in Mainland of China.

**Methods:**

This study systematically collected a total of 192 cases of children's clothing-related injuries reported in the news reports and medical literatures published by the Chinese media before December 2017. A content analysis of these cases was conducted to obtain information on the characteristics of these injuries.

**Results:**

Cases of clothing-related injuries were most common in children aged 3 to 6 years (54.7%); the ratio of the number of boys to the number of girls in the 192 cases was 1.5: 1. In addition, approximately 51% of the cases occurred in the region of East China. The most common location of the incidents was in the home (35%). Furthermore, the zippers are the most common cause of injuries (15.1%), and the most common injured body part was the genital organs (29.2%). Besides, there were 24 cases reported death related to clothing injuries (13%). Moreover, the locations where the injuries occurred, the reasons for the occurrence of the injuries and the injured body parts differed significantly by gender and age (*P* < .05).

**Conclusions:**

Our findings suggest that future unintentional injuries could be prevented by implementing 2 major initiatives: improving the promotion of the safety of children clothing design in China and educating consumers on the potential risk associated with children's clothing.

## Abstract 2

Supplemental Digital Content is available in the text

## 1 Introduction

Children's clothing has a significant impact on children's health, safety, and well-being. These outcomes can be affected not only by the type of clothes that children wear but also how these clothes are worn. A child's age is also an important factor in determining how children's clothing may affect their comfort and safety. Considering children's activity and defects in clothing, children's clothing may cause a large number of potential mechanical hazards, which may affect the safety of children, such as choking, swallowing, strangulation, entrapment, entanglement, feelings of being trapped, snagging, tripping, falling, and sliding.

Young children often swallow buttons and other decorative foreign bodies, inhale them or push them into body cavities such as the nose and ear and mouth. For example, according to the 1995 statistics of the British Household accident monitoring system, there are 154 registered cases of children's accidents and pediatric emergency cases in designated hospitals involving children and their clothing.^[1]^Additionally, the US Consumer Product Safety Commission reported 12 deaths and 27 near-strangulation cases related to playground equipment between 1985 and 1994. At least 25 of these strangulation cases involved in children's clothing. ^[2]^ Also, in Sweden, during a 10-year period, 5 of the 73 cases of accidental mechanical asphyxiation involved children's clothing.^[3]^ Furthermore, Pamela Norum et al^[4]^ indicated that China's infant products reached a higher proportion of recalls in 2011, with 32.77% or 78% being recalls of infants’ and children's apparel, which was the same as the proportion of deaths/injuries related to strangulation or fall.^[4]^

In recent years, with the gradual popularization of the Internet, consumers have begun shopping children's clothing on online platforms. The 3 largest online shopping platforms in China are Jingdong (JD), Taobao (TB), and Tian mao. Under the rapid development of children's clothing network platform Market in China,^[5]^ the quality of some children's clothing online has become a major concern, especially with the fact growing e-commerce market in China.^[5]^ According to the results reported by the Beijing City Consumer Association on online shopping for children's clothes, nearly 40% of the children's/infants’ clothing products, including those sold via the JD and TB online platforms are unsafety. Furthermore, nearly 31 brands of children's clothes were reported to be of inferior quality.^[6]^

In China, few studies have investigated children's clothing-related injuries, and no epidemiological information is available on injury prevention and control in the pediatric population. As children are more vulnerable to be injured than adults due to their age and activity, it is necessary to systematically collected the medical literature to analyze potential hazards of children's clothing, and to provide some useful information for children injuries prevention. Therefore, the purpose of this study was to provide a description of children's clothing-related injuries based on information about such incidents reported by the media and related journals.

## 2 Methods

### 2.1 Ethical statement

On this study, it was not necessary to obtain institutional review board's approval due to the use of publicly accessible data of published cases and the project being not involved patient consent.

### 2.2 Case identification and search strategy

We conducted an extensive search for relevant news reports of incidents related to children's clothing on search engines (Google Scholar, Baidu, SOHU News, Tencent News, and Sina News) as well as in the 3 main Chinese Scientific Journals (Wanfang, and CQVip). The extensive search targeted relevant information published not later than December 31, 2017. In addition, only articles published in the Chinese language were selected and the search terms for retrieving the relevant information were based on the subject terms, keywords, and titles. Therefore, a combination of MeSH terms was used ((“children's clothing” or “children's garments” or “infants’ apparel” or “children dress,” “drawstrings,” “synthetic skirt,” “scarves,” “shoes,” “trousers,” “open-seat pants,” “gloves,” “socks,” “trousers with metal zippers,” “coats with metal zippers,” “decorative items on clothing”) and (“injuries,” “wounds” or “casualties, accidents or safety”)) to retrieve relevant information. In addition, the reports of relevant clothing-related accidents associated to printed media in mainland of China were also collected.

### 2.3 Case selection and data extraction

Initially, retrieved information was screened by examining the title, abstract or background. Children's clothing-related injuries were considered eligible for this study if they met the following criteria:

(1) they were reported during the study period in China;
(2) they were overwhelming evidences of threat to the safety of children, such as suffocation, choking, aspiration, asphyxiation, casualty, disability, or death;
(3) they were abrasions, contusions, spinal cord injuries, fractures, falls, strangulation injuries related to long drawstrings on garments, scarves, and ties;
(4) they were genital and eyelid-related injuries;
(5) they were shoe-related injuries;
(6) they were burns involved clothing;
(7) they were skin allergic reaction;
(8) they were injury incidents in mainland of China;
(9) they were incidents related to improper use of children's clothing that resulted in the child being sent to the hospital; and
(10) they were media-reported cases of death of a victim who had been diagnosed by a general practitioner or had a record of a past incident of injury due to children clothing.

**Figure 1:**
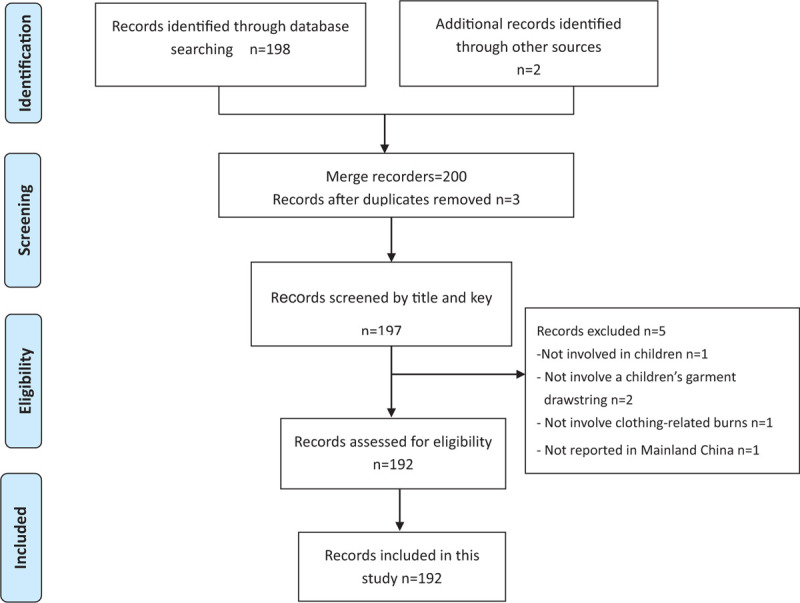
Flow diagram of included/excluded studies. A total of 200 documents were found in the initial search. After removing duplicates, reading titles, abstracts and background informations, 192 eligible cases were included and analyzed. Figure 1 Flow diagram of included/excluded studies. A total of 212 documents were found in the initial search. After removing duplicates, reading titles, abstracts and background informations, 192 eligible cases were included and analyzed.

A description of each of the preceding cases can be found in the additional Supplement File 1: Table S1. Based on the preceding inclusion criteria, 2 independent authors (SXC and XJC) extracted the following data and tabulated them in Excel 2010, that is, the year, month, and time of the incident; and information about the child, such as name, age, gender, province where the incident occurred, place where the incident occurred, reason for the incident, injury outcomes and media resources. If the opinions of the 2 independent authors were consistent regarding to a clothing-related injury, the incident was either included or excluded accordingly. Any disagreements between them were resolved through consensus or arbitration by the third reviewer (HLX). Audits and checks of the keyword search were carried out to ensure the completeness of the case identification procedures. A detailed search flowchart is shown in Figure 1.

## 3 Statistical analyses

Statistical analysis was performed in SPSS version 20.0 (SPSS, Inc., Chicago, IL). Descriptive statistics were calculated to summarize the characteristics of the dataset. The Chi-square test was used to examine the distributions of outcomes of interest across the categorical variables. Two-sided *P*-values were considered statistically significant at *P* < .05. A statistical map showing the regional distribution of media-reported incidents of children injuries related to clothing was plotted using ArcGIS 10.2 statistical software (Environmental Systems Research Institute, Inc Redlands, State of California, USA).

## 4 Results

### 4.1 General characteristics

In this study, 192 incidents involving victims of children clothing-related injuries were identified during the study period. There were 115 boys (59.1%) and 77 girls (40.1%), representing the ratio of 1.5 boys to 1 girl. The children's ages ranged from 0 to 17 years, and the mean (standard deviation) age of the children was 4.86 (2.49) years. The majority of the children were aged 3 to 6 years (54.7%). Table 1 displays more detail about the characteristics of the sample.

**Table 1:**
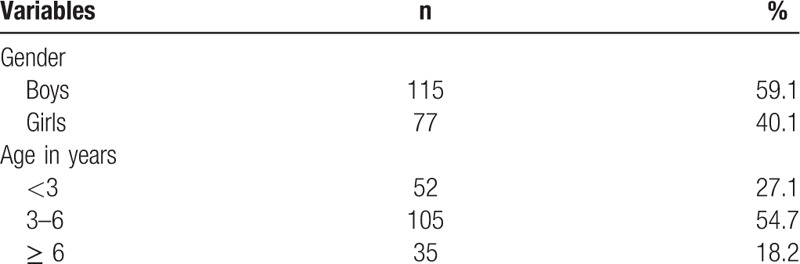
Sample distributions of gender and age groups (n = 192).

### 4.2 Case details as reported by the media

#### 4.2.1 Locations of the occurrence of clothing-related accidents

Of the 192 incidents, only 140 had their locations reported. In this regard, the majority of the injuries occurred in the home (35%, 49/140), followed by kindergartens (20%, 28/140), then in the street (19.2%, 27/140). However, 52 cases (27.1%) were not described in detail locations. There were significant differences in the location of clothing-related accidents by age and gender (*P* < .05, Monte Carlo test). Detailed results are displayed in Table 2.

**Table 2:**
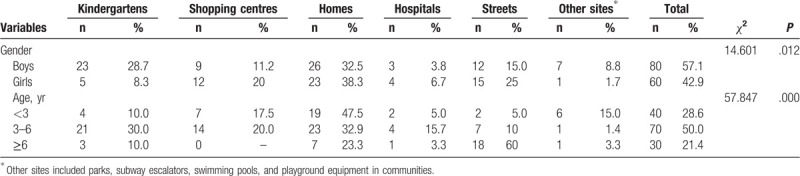
Distribution of locations of occurrence of children's clothing-related injuries across their gender and age groups.

#### 4.2.2 Reasons for the occurrence of victims’ accidents

All 192 cases reported the cause of the accidents. The metal zippers involved incidents (15.1%) was the most common cause of injuries (15.1%, 29/192), followed reported incidents were by scarves/shawls and open-seat pants (13.5%, 26/192). Then common reason was that the children's neck is entangled by the long drawstrings with children's garments (13%, 25/192). There were significant differences in the reasons for clothing-related accidents by age and gender (*P* < .05, Monte Carlo test). These results are displayed in Table 3.

**Table 3:**
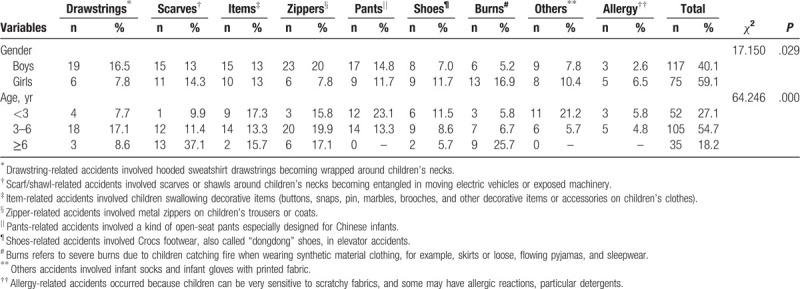
Distribution of reasons for the occurrence of children's injuries across their gender and age groups.

#### 4.2.3 Distribution children's injured body parts

Again, the injured body parts were reported for each of the 192 cases. Thus, the most common injured body parts were genital organs (29.2%, 56/192); followed by necks (27.1%, 52/192), then other body parts such as eyes, nose, ears, mouth, and throat (13.5%, 26 /192). There were significant differences in injured body parts by gender and age (*P* < .05, Monte Carlo test). These results are displayed in Table 4.

**Table 4:**
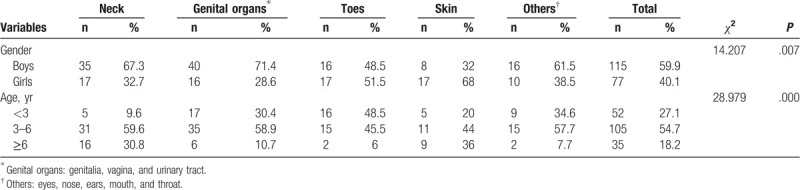
Distribution of children's injured body parts across their age groups and gender.

#### 4.2.4 Regional distribution of children's clothing-related injury cases

**Figure 2:**
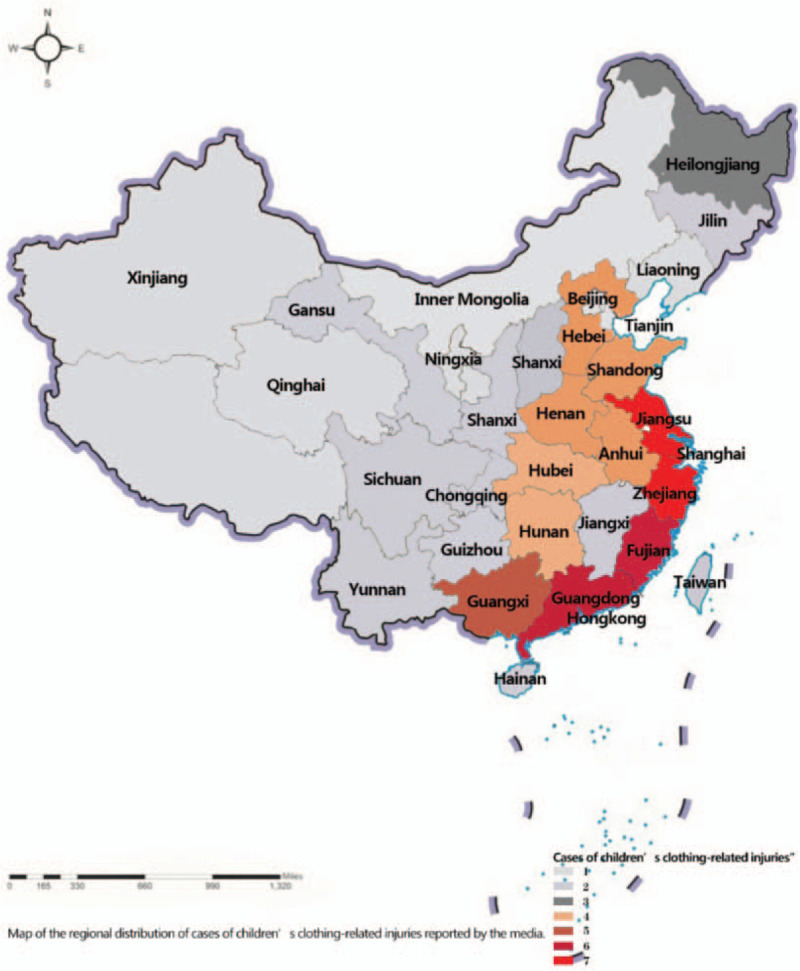
Regional distribution of cases of children's clothing-related injuries reported by the media.

Of the 192 cases, the location of 6 cases was not known. Therefore, 186 cases were distributed among 29 locations (Provinces or Municipalities), and the 4 Provinces with largest numbers of cases were located in the coastal areas of mainland of China: Jiangsu Province (11.8%, 22/186), Zhejiang Province (10.8%, 20/186), Fujian Province (7.5%, 14/186), and Guangdong Province (7%, 13/186). On the other hand, Tianjin Municipality, Yunnan Province, Guizhou Province, Hainan Province, Hong Kong, Taiwan, Xinjiang, and Inner Mongolia had relatively a few cases reported as each area had only 1 case (0.5%, 1/186). With regard to the 8 geographical areas of mainland of China, there was a regional high concentration of reported cases in East China (Jiangsu, Zhejiang, Fujian, Shanghai, Guangdong, Shandong, Anhui, and Jiangxi), which they were 95 cases (51%, 95/186). The detailed regional distribution characteristics are shown in Figure 2.

#### 4.2.5 Accidental death cases reported by media

In the current study, 24 deaths cases (13%, 24/192) were identified. A significant relationship was observed between death and accidental suffocation. Most cases were associated with the entanglement of a drawstring (belts, ties, and drawstrings in sweatshirts, hooded sweatshirts, and waist belts). In these cases, some children were strangled by the ropes of their own clothes when they were playing on the slide. For example, a 4-year-old girl (in Guangxi Zhuangzu autonomous region) was strangled because the drawstring on the hood of her jacket was caught in a U-shaped gap at the top of the slide. And because the weight of the girl's body pulled the drawstring taut around her neck. When the supervisors noticed her, the girl has not breathing. Another case involved a 10-year-old boy in Shanghai being crushed to death when a drawstring on his jacket was caught in a crack of a door of a moving bus. Furthermore, a 6-year-old boy in Wuhan, Hubei Province, was wearing a hooded garment, when 2 other children pulled his garment by the drawstrings, which this action was resulted in fatal strangulation. Also, there were 2 cases (8.3%, 2/24), children swallowed decorative items or placed objects in their mouths, which ultimately resulted in serious suffocation events. Moreover, there were 7 incidents of fatal asphyxiation which the main reason was that scarves around children's necks became caught in electric vehicle wheels. The detailed background descriptions are summarized in Table 5.

**Table 5:**
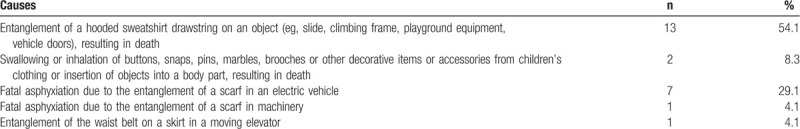
Causes of death related to children's clothing (n = 24).

## 5 Discussion

### 5.1 Primary findings

This is the first systematic analysis for the epidemiological characteristics of children's clothing-related injuries cases which was reported by the media in mainland of China.

In terms of demographic characteristics, boys were experienced more clothing-related injuries than girls, most children's clothing-related incidents occurred in children aged 3 to 6 years (54.7%); thus, this age group is considered to be very vulnerable with a serious risk of unintentional injury, hence at a higher risk of injuries related to clothing.

A previous similar study also showed that boys experienced more unintentional injuries than girls. Generally, children are more prone to get injured because they are young and very active with little experience. As a way of minimizing injuries among children, it is necessary to analyze the potential hazards of children's clothing, identify direct and indirect impacts of these potential hazards, and identify ways to control these hazards in terms of clothing design, manufacturing, and inspection.^[7]^

Also, incidents of children's injuries related to children's clothing occurred in the regions of East and South China. This result shown that people's consumer-level factors might affect the characteristics distribution of children's clothing-related accidents among children populations. Furthermore, in terms of circumstances surrounding the occurrence of the injuries, 13.5% of the accidents involved entanglement of a scarf in an electric vehicle or machinery, whereas 13% of the accidents involved the entanglement of a garment drawstrings that were too long. It is also possible that overuse of drawstring clothing or improper use of scarf contributed to these incidents. Unfortunately, in 8.9% of the cases, children's fingers or toes were injured due to Crocs shoe-related accidents on elevators in shopping centers or supermarkets. In addition, the most common injuries occurred at the home (35%). This result suggested that more injury prevention interventions should be developed to prevent children's injuries in their homes.

Additionally, this study has demonstrated that a relatively high proportion (15.1%) of zipper-related genital injuries occurred due to the use of trousers in children. Child factors that contribute to childhood injury are developmental, physical, and behavioral in nature.

Besides, many parents of children are not awareness of some children's/infants’ apparels being unsafe for younger children, and significantly increase the injury risk for pre-schoolers by allowing them to use these clothing.^[8,9]^ For example, a survey conducted in 2013 by the General Administration of Quality Supervision and Inspection People's Republic of China revealed that the majority of Chinese consumers were not concerned about the risk posed by children's clothing in the Chinese market and that only 10% had addressed the potential hazards.^[10]^ Despite the safety information provided on each item of clothing purchased by consumers, parents are often not aware of the potential hazards. Another possibility with lack of awareness is that people purchased children's clothing is in the lower-end markets in rural areas instead of departmental stores. Any caregivers are conscious of low discounted prices or fashions and ignore or are not aware of the potential hazards of unsafe children's clothing. It is important to educate parents and encourage caregivers to select safe clothing for their children.

In this study, it's worth noting that 19 victims caught fire because of their clothes on fire and suffered burn injuries. For instance, a 7-year-old left-behind children (girl) experienced extensive burns when her skirt made of synthetic material caught fire while cooking dinner in the kitchen of her home in Bozhou State, Anhui Province on June 10, 2017. A similar study was reported in Canada.^[11]^ Low socioeconomic status, poor living conditions, and poor literacy have been associated with a risk of burn injury.^[12–15]^ Nevertheless, a standard for children's clothing flammability risk has not been adopted in China. Our research suggests that clothing-related burn risks can be reduced by stronger government regulations and by educating caregivers to let children dress less flammable fabrics at home and avoid the use of unsafe cooking appliances, especially in overcrowded kitchens.^[16]^

As regards drawstring garment requirements, on July 1, 2016, the Chinese government issued a product safety standard (GB31701-2015) to address drawstrings on children's upper outerwear garments to prevent children's resulting from drawstring entanglement.^[16]^ The most recent revision to the standard was made 1 year later in June 2017 in an attempt to reduce various risk of strangulation associated with different types of children's clothing. Unfortunately, a fatal accident still occurred in a school in Jiangan District in Wuhan, Hubei Province, on November 22, 2017. Therefore, to address these problems, detailed child-related clothing safety recall strategies should be strengthened to reduce such potential hazards and to ensure the safety of children's clothing in China. In the United States of America (USA), a recommended voluntary standard was outlined for the requirements with drawstrings in children's clothing that was adopted from 1997 to 2009. After the implementation of voluntary standards, it effectively reduced the deaths cases caused by strangulation involved the entanglement of children's and teenagers’ jackets with drawstrings. The drawstring requirements established in the voluntary standard were associated with a 90.9% (95% confidence interval, 83.8%–96.1%) reduction in drawstring-related mortality.^[17]^ In Canada, Petruk et al^[18]^ reported cases of deaths caused by strangulation involving entanglement of children's clothing. The authors of that study recommended the removal of all cords, straps, and knobs from children's clothing.

### 5.2 Implications

This study provides an understanding of the characteristics of children's clothing-related injuries in mainland of China, and offers a medical, and scientific basis for the prevention and intervention strategies of children's clothing related to injuries. Although accidents involving children's clothing are far less common than other types of accidents, they can cause trauma and death in various ways, such as burns from flammable materials clothing and strangulation from the entanglement of drawstrings with hood clothing.^[11,19]^ Hooded sweatshirt drawstring-related casualties are rare but devastating, especially they occur in environments that most children consider to be safe. In this study, the proportions (13%) of strangulation and entrapment injuries associated with drawstrings in clothing were higher than the proportions of injuries caused by other children's products. For example, Heather Beach reported 9.9% of the victims being head injuries associated with Heelys.^[20]^ Therefore, this should warrant a discussion on public health measures to avoid injuries related to clothing in children.

### 5.3 Limitations

There are several limitations that should be acknowledged in this study. First, all data were obtained from the media reports and journal databases, hence may be prone to selection bias because we could not verify that all patients with clothing-related incidents were included. Second, some media reviews might have provided reported data that were not accurate or complete; therefore, a possibility of reporting bias cannot be ignored when interpreting the findings of this study.

## 6 Conclusions

Despite its shortcomings, this study provides an in-depth understanding of the main characteristics of the risk of children's clothing-related injury in mainland of China. Accordingly, our findings suggest that future unintentional injuries could be prevented by implementing 2 major initiatives: improving the promotion of the safety of children clothing design in China and educating consumers on the potential risk associated with children's clothing.

## Acknowledgments

We take the opportunity to sincerely thank the anonymous reviewers for their thoughtful and meaningful comments. This study was supported by the School Foundation of Guizhou Minzu University (Grant No. GZMU ([2019]YB05)).

## Author contributions

**Conceived and designed the analysis:** Sixiang Cheng, Huilan Xu.

**Conceptualization:** Sixiang Cheng, Huilan Xu.

**Data curation:** Xunjie Cheng

**Formal analysis**: Sixiang Cheng.

**Funding acquisition:** Sixiang Cheng.

**Methodology:** Sixiang Cheng. Xunjie Cheng.

**Performed the analysis:** Sixiang Cheng, Xunjie Cheng.

**Software:** Sixiang Cheng, Xunjie Cheng.

**Supervision:** Huilan Xu.

**Writing – original draft:** Sixiang Cheng.

**Writing – review and editing:** Sixiang Cheng, Atipatsa Chiwanda Kaminga, Huilan Xu.

**Wrote the paper:** Sixiang Cheng.

## Supplementary Material

Supplemental Digital Content
