# Supplementary material for: An analysis of children's clothing-related injuries cases reported by the media in mainland of China from 2003 to 2017
Source: Medicine (Baltimore). 2020 Feb 28;99(9):e19305. doi: 10.1097/MD.0000000000019305 (PMC7478676; doi:10.1097/MD.0000000000019305)
Supplement: Supplemental Digital Content [file medi-99-e19305-s001.docx]

**Table S1 Characteristics of included cases**

| **No** | **Year/month /data** | **Provinces** | **Ages** | **Gender** | **Locations** | **Reasons descriptions** | **Outcomes** | **Data sources** |
| --- | --- | --- | --- | --- | --- | --- | --- | --- |
| 1 | 2003/2/17 | Henan | 5 | boy | kindergarten | Drawstrings-related with clothing | death | http://www.sohu.com/a/210026468_801371 |
| 2 | 2005/6/3 | Henan | 5 | boy | street | wear open- pants | burning | http://www.sina.com.cn |
| 3 | 2006/2/23 | Hebei | 17 | boy | street | shoes-related fall | brain injuries | http://www.sohu.com/a/156925480_799858 |
| 4 | 2006/11//3 | Shanxi | 3 | boy | street | worn open- pants and his genital injured by dog bites | genital injured | http://www.sina.com.cn |
| 5 | 2007/1/6 | Guangxi | 4 | girl | kindergarten | drawstrings-related with clothing | death | http://blog.sina.com.cn/s/blog_c1e7e.html |
| 6 | 2007/10/22 | Fujian | 3 | boy | kindergarten | drawstrings-related with clothing | asphyxiation | www.hxnews.com |
| 7 | 2007/10 | Hongkong | 3 | girl | Shopping centers | Crocs shoes involved the elevator | toes were injured | http://roll.sohu.com/20120710/n347763819.sht |
| 8 | 2009/1/1 | Hubei | 6 | girl | home | clothing catch on fire | burning | Sichuan workers daily |
| 9 | 2009/5/20 | Hunan | 3 | boy | shopping centers | open backed pant and fall in elevators | genital injury | www.cssqt.com |
| 10 | 2009/5/20 | Fujian | 2 | boy | street | wear open- pants | genital injury | Xiamen evening news |
| 11 | 2009/7/1 | Hainan | 5 | girl | hospital | swallow decorative items with clothing | mouth inured | The journal of Chinese fiber inspection china,201414(4) |
| 12 | 2009/11/16 | Zhengzhou | 5 | girl | hospital | down jacket-involved allergy | nose | The journal of Family practice, 2010(1) |
| 13 | 2010/1/10 | Hubei | 4 | boy | school | mental zipper with trouser | genital injury | http:/www .news.sohu.com |
| 14 | 2010/8/6 | Shandong | 4 | boy | home | cartoon short sleeves | skin allergic reactions | Yantai evening newspaper |
| 15 | 2010/12/22 | Zhejiang | 4 | boy | kindergarten | drawstrings-related with clothing | death | Wenzhou business paper |
| 16 | 2010/12/29 | Hebei | 10 days | boy | home | socks with fibers involved | fingers | http://www.chinanews.com |

**Table S1 Characteristics of included cases(continued)**

| **No** | **Year/month /data** | **Provinces** | **Ages** | **Gender** | **Locations** | **Reasons description** | **Injury outcomes** | **Data sources** |
| --- | --- | --- | --- | --- | --- | --- | --- | --- |
| 17 | 2011/2/19 | Henan | <1y | boy | home | sock involved | toes injured | http://www.sina.com.cn |
| 18 | 2011/2/24 | Yunnan | 6 | girl | home | clothing catching on fire | burning | https://www.bjnews.com.cn |
| 19 | 2011/2/24 | Hebei | 9 | boy | home | clothing catching on fire | burning | Yanzhao Metropolis Daily |
| 20 | 2011/2/25 | Shandong | 4 | Girl | kindergarten | drawstrings | death | http://www.sohu.com/a/210026468_ |
| 21 | 2011/4/1 | Jiangxi | <3y | girl | kindergarten | drawstrings | death | http://www.sina.com.cn |
| 22 | 2011/4/18 | unknown | 6 | girl | drawstrings | drawstrings | death | https://www.360kuai.com |
| 23 | 2011/4/26 | Zhejiang | 4 | girl | swimming poor | jacket | skin allergic reactions | [www.ytse](http://www.ytse) evening papers |
| 24 | 2011/7/9 | Zhejiang | 5 | boy | supermarket | Crocs shoes | two figures were cut off | www.yangtse.com |
| 25 | 2011/7/11 | shanghai | 3 | girl | supermarket | Crocs shoes | toe | http://news.sina.com.cn/c/2011-07 |
| 26 | 2011/7/22 | Hunan | 6 | boy | hospital | worn open- pants | his genital was bit by a horse | www.bj.chinanews.com |
| 27 | 2011/8/16 | Hebei | 5 | girl | home | skirt catch on fire | burning | Yanjing evening papers |
| 28 | 2011/10/18 | Jiangxi | 1 | boy | home | coat catch on fire | burning | https://www.haodf.com/wenda/xchin |
| 29 | 2011/12/10 | Guangxi | 4 | boy | kindergarten | drawstrings | almost asphyxiation |  |
| 30 | 2011/11/30 | Hubei | 5 | girl | supermarket elevator | the rope entanglement with his skirt | almost asphyxiation | Wuhan evening news |
| 31 | 2011/11/29 | Fujian | 4 | boy | unstated | mental zipper with trouser | genital injured | metropolis papers |
| 32 | 2012/2/10 | unstated | 6 | girl | playground | scarf-related injury | almost asphyxiation | https://www.2500sz.com |
| 33 | 2012/2/28 | Jiangsu | 6 | girl | elevator | Crocs shoes-related | The knee joint being injured | https://www.news.subaonet.com |
| 34 | 2012/3/1 | Jiangsu | 8 | boy | street | roller skates-related | fall, knee joint being injured | https://www.xinhuanet.com |
| 35 | 2012/3/7 | Zhejiang | 4 | boy | unstated | mental zipper struck with his trousers | genital injured |  |
| 36 | 2012/3/19 | Sichuan | 4 | girl | shopping center | high heels shoes-related | fracture | chongqing evening papers |

**Table S1 Characteristics of included cases(continued)**

| **No** | **Year /month/data** | **Provinces** | **Years** | | **Gender** | **Locations** | **Reasons description** | **Injury outcomes** | **Data sources** |
| --- | --- | --- | --- | --- | --- | --- | --- | --- | --- |
| 37 | 2012/4/1 | Henan | | 4 | girl | street | elevator of a supermarket | fatal asphyxiation | https://www.xdkb.net |
| 38 | 2012/5/19 | Niaoling | | 5 | girl | hospital | swallow decorative items with clothing | mouth | http://dzb.hxnews.com/2013-03/20/content_97311.htm |
| 39 | 2012/5/30 | Shandong | | 1 | boy | home | clothing catch fire | burning | Qilu evening papers |
| 40 | 2012/5/30 | Jiling | | 10 | girl | home | skirt catch fire | burning | http://jl.sina.com.cn/news/cssd/2015-07-15/detail-ifxfaswi403 |
| 41 | 2012/6/6 | Guangxi | | 6 | girl | hospital | clothing-related allergic reactions | skin allergic reactions | http://www.sina.com.cn |
| 42 | 2012/6/22 | Zhejiang | | 7 | boy | unstated | mental zipper with trousers | genital injured | http://www.sina.com.cn |
| 43 | 2012/7/1 | Guangdong | | 6 | boy | supermarket | Crocs shoes | his foot was struck by escalator | Beijing youth daily |
| 44 | 2012/7/20 | Zhejiang | | 5 | boy | supermarket | Crocs shoes | figures were injured | Beijing youth daily |
| 45 | 2012/9/15 | Zhejiang | | 2 | boy | unstated | mental zipper with trousers | genital injured | Journal of Zhejiang trauma surgery |
| 46 | 2012/10/1 | Guangdong | | 5 | boy | elevator | Crocs shoes involved | toes were injured | Beijing youth daily |
| 47 | 2012/11/1 | unstated | | 3 | girl | unstated | push marble into her throat | choking | http://www.sina.com.cn |
| 48 | 2012/11/5 | Hunan | | <1y | girl | home | socks thread involved | toe muscle damage | http://control.blog.sina.com.cn/myblog/htmlsource/blog_notopen.php |
| 49 | 2012/11/12 | Sichuan | | <1y | boy | home | glove thread involved | figures were injured | www.voc.com.cn |

**Table S1 Characteristics of included cases(continued)**

| **No** | **Year /month/data** | **Provinces** | **Years** | **Gender** | **Locations** | **Reasons** | **Injury outcomes** | **Data sources** |
| --- | --- | --- | --- | --- | --- | --- | --- | --- |
| 50 | 2012/11/20 | Zhejiang | 5 | boy | hospital | push ornaments (12 pieces) into her ear | right ear loss of hearing | http://www.wendu.com |
| 51 | 2012/11/29 | Zhejiang | 5 | girl | hospital | push decorative into her eye | right ear loss of hearing | http://www.weather net |
| 52 | 2012/12/2 | Hubei | 5 | girl | supermarket | scarf entanglements | fatal asphyxiation | Wuhan evening daily |
| 53 | 2012/12/2 | Guizhou | <1y | boy | park | wear open-backed pants | by dog bit injured | hsb.hspress.net |
| 54 | 2012/12/8 | Jiangsu | <1y | girl | home | gloves thread wrapped | figure injured | http://www.babytree.com/communit |
| 55 | 2013/2/20 | Sichuan | 5 | boy |  | new clothes | cough, asthma | Chongqing morning papers |
| 56 | 2013/3/20 | Fujian | <1y | boy | home | accidentally swallowed a small pin holding the amulet on the chest into the mouth | his throat were injured | Haixia metropolis daily |
| 57 | 2013/3/27 | Fujian | <1y | boy | home | socks thread involved his figure | toes were injured | http://www.99 healthy network |
| 58 | 2013/4/1 | unknown | <1y | boy | home | swallowed a decorative items on the coat into throat | death | http://jl.sina.com.cn/news/cssd/2015-07-15/detail-ifxfaswi403 |
| 59 | 2013/6/22 | Guangdong | 7 | boy | street | shoes | traffic accidents | [12 traffic net](http://www.122.cn/) |
| 60 | 2013/4/1 | Zhejiang | 3 | boy | street | Crocs shoes involved | toes injured |  |
| 61 | 2013/5/1 | Fujian | 4 | boy | unstated | mental zippers involved injury | genital injured | The journal of Medical world |
| 62 | 2015/8/4 | Fujian | 5 | boy | home | mental zippers related | genital injured | The journal of medical source world |
| 63 | 2013/5/28 | Jiangsu | 5 | boy | unstated | clothing drawstrings entanglement with his neck | fatal asphyxiation | Suzhou evening papers |
| 64 | 2013/6/5 | Zhejiang | 2 | boy | hospital | mental zippers was struck with trouser | genital injured | Zhejiang online |

**Table S1 Characteristics of included cases(continued)**

| **No** | **Year /month/data** | **Provinces** | **Years** | **Gender** | **Locations** | | **Occurrence reasons** | **Injury outcomes** | **Data sources** |
| --- | --- | --- | --- | --- | --- | --- | --- | --- | --- |
| 65 | 2013/7/9 | Zhejiang | 5 | boy | elevator | | Crocs shoes involved | toes injured | Beijing young daily |
| 66 | 2013/7/27 | Hebei | 8 | girl | hospital | | push decorative item with her clothing into her ears | ear disabled | Hebei youth daily |
| 67 | 2013/8/4 | Fujian | 9 | boy | unstated | | new clothing | skin allergies | http://www.sohu.com/a |
| 68 | 2013/9/1 | Hebei | 4 | girl | kindergarten | | decorative item with her clothing | loss of hearing in the ear | hzdaily papers |
| 69 | 2013/9/17 | Jiangsu | 8 | girl | street | | the skirt was entanglement in the wheel | cervical injured | https://www.people.com.cn- |
| 70 | 2013/9/18 | Fujian | 5 | boy | hospital | | open-backed pants | genital inured | Southeast net |
| 71 | 2013/9/22 | Guangdong | 5 | girl | kindergarten | | clothing drawstrings entanglement his neck | death | Yangcheng evening daily |
| 72 | 2013/9/23 | Jiangsu | 2 | boy | unstated | | decorative item with her clothing | throat were injured | Jinling evening daily |
| 73 | 2013/9/27 | Jiangxi | 6 | boy | kindergarten | clothing drawstrings entanglement his neck | | fatal asphyxiation | http://www.sohu.com/a/210026468_801371 |
| 75 | 2013/7//9 | Guangzhou | 2 | boy | kindergarten | decorative item with her clothing | | head was injured | Jinling evening daily |
| 76 | 2013/9/26 | Henan | 3 | boy | kindergarten | drawstrings-related | | death | Dahe daily papers |
| 77 | 2013/11/27 | Jiangxi | 3 | boy | kindergarten | drawstrings-related | | fatal asphyxiation | http://www.sohu.com/a/210026468 |

**Table S1 Characteristics of included cases(continued)**

| **No** | **Year /month/data** | **provinces** | | **Years** | **Gender** | | **Locations** | | **Occurrence reasons** | **Injury outcomes** | **Data sources** |
| --- | --- | --- | --- | --- | --- | --- | --- | --- | --- | --- | --- |
| 78 | 2013/12/20 | Jiangsu | | 2 | boy | | home | | dragged pins with her clothing his throat | death | Jiangsu evening news |
| 79 | 2013/10/24 | Guangdong | | 6 | boy | | unstated | | mental zipper with his trousers | genital injured | http://www.sina.com.cn |
| 80 | 2013/11/1 | Zhejiang | | <1y | boy | | street | | glove involved | toes were injured | http://www.sina.com.cn |
| 81 | 2013/12/18 | Sichuan | | 1 | boy | | home | | open-backed pants | burns | http://www.hualong.com.cn |
| 82 | 2014/1/11 | Henan | | 7 | girls | | street | | scarf-related injuries by electric vehicle wheels | strangulation | Henan business papers |
| 83 | 2014/2/10 | Xinjiang | | 8 | girl | | street | | scarf-related injuries by exposed machinery | death | http://sports.iqilu.com/outdoor/20140210/1858781.shtml |
| 84 | 2014/2/16 | Sicuan | | 9 | boy | | street | | clothing- elevators | death | Ningxia TV |
| 85 | 2014/2/18 | Jiangsu | | 3 | boy | | kindergarten | | drawstring clothing | strangulation | Yangzi river evening papers |
| 86 | 2014/3/1 | Fujian | 3 | | girls | unstated | | swallow decorative items on clothing | | ears be injured | http://news.163.com/14/0301/10/9M8C29 |
| 87 | 2014/3/11 | Zhejiang | 3 | | girls | kindergarten | | push buttons into her vagina | | strangulation | China jiangsu net |
| 88 | 2014/3/13 | Zhejiang | 10 | | girls | street | | scarf-related injuries by electric vehicle wheels | | strangulation | http://www.sohu.com/a/8411190_111870 |
| 89 | 2014/3/18 | Zhejiang | 3 | | girls | street | | scarf wrapped by bike | | asphyxiation | http://blog.sina.com.cn/s/blog_af73ff160101sb3s.html |
| 90 | 2014/3/27 | Jiangsu | 9 | | girls | unstated | | mental zipper with coat | | Eye were injured | Yantai daily paper |

**Table S1 Characteristics of included cases(continued)**

| **No** | **Year /month/data** | **Provinces** | | **Years** | **Gender** | | **Locations** | | **Occurrence reasons** | **Injury outcomes** | **Data sources** |
| --- | --- | --- | --- | --- | --- | --- | --- | --- | --- | --- | --- |
| 91 | 2014/4/15 | Hebei | | 8 | girls | | school | | mental zipper with coat | eye were injured | http://news.sina.com.cn/.shtml |
| 92 | 2014/4/21 | Hebei | | 6 | girls | | unstated | | synthetic skirt | burning | Chongqing evening daily |
| 93 | 2014/5/5 | Sicuan | | 9 | girls | | home | | skirt catch on fire | burning |  |
| 94 | 2014/5/19 | Niaoning | | 4 | boy | | unstated | | new clothing | skin allergies | Huasheng morning papers |
| 95 | 2014/6/10 | Jiangsu | | 4 | boy | | home | | clothing fire | burning | People daily net |
| 96 | 2014/8/1 | Hunan | | 4 | girls | | shopping center | | Crocs shoes involved elevator | fracture | Beijing young daily |
| 97 | 2014/8/1 | Shandong | | 4 | girls | | shopping center | | Crocs shoes involved elevator | figure injured | baijiahao.baidu.com/s?id=1 |
| 98 | 2014/9/13 | Anhui | | 5 | boy | | school | | mental zipper involved | genital injured | http://news.ifeng.com/c/404 |
| 99 | 2014/7/9 | Zhejiang | 5 | | boy | shopping center | | Mental zipper involved | | genital injured | http://roll.sohu.com/20150804/n418171112. |
| 100 | 2014/10/4 | Shanghai | 3 | | boy | elevators | | ties with her shoes | | figure injured | https://baike.1688.com/doc |
| 101 | 2014/10/11 | Henan | 5 | | boy | unstated | | mental zipper involved | | genital injured | Zhengzhou evening papers |
| 102 | 2014/11/21 | Sichuan | 10 | | boy | street | | scarf entanglements by vehicle wheels | | injured | Chengdu business papers |
| 103 | 2014/11/25 | Shandong | 4 | | girl | street | | scarf- entanglements by vehicle wheels | | asphyxiation | https://www.bzcm.net/ |
| 104 | 2014/11/1 | Hebei | 7 | | boy | street | | scarf- entanglements by vehicle wheels | | asphyxiation | https://blog.sina.com.cn/s/blog_.html |
|  |  |  |  | |  |  | |  | |  |  |
| **Table S1 Characteristics of included cases(continued)** | | | | | | | | | | | |
| **No** | **Year /month/data** | **Provinces** | | **Years** | **Gender** | | **Locations** | | **Occurrence reasons** | **Injury outcomes** | **Data sources** |
| 105 | 2014/12/1 | Nei Mongol Autonomous | | <1y | boy | | street | | scarf- entanglements by vehicle wheels | death | Business daily |
| 106 | 2014/12/2 | Tianjing | | 10 | girl | | street | | scarf-entanglements by vehicle wheels | spine injuries | Dahe helth daily |
| 107 | 2014/12/5 | Hebei | | 10 | girl | | street | | scarf- entanglements s by vehicle wheels | spine injuries | Health.zjol.com.cn |
| 108 | 2014/12/11 | Shanxi | | 3 | girl | | street | | scarf- entanglements by vehicle wheels | spine injuries | Nanjing morning papers |
| 109 | 2014/12/26 | Hunan | | 5 | boy | | kindergarten | | mental zipper involved | genital injured | https://.Hnsina.com.cn |
| 110 | 2015/6/25 | Guangdong | | 7 | boy | | street | | shoes-related fall | fracture | 122 traffic net |
| 111 | 2015/1/8 | Shanghai | | 10 | boy | | street | | bus dragged his clothing with drawstrings | death | www.eastday.com |
| 112 | 2015/1/14 | Shandong | | 3 | boy | | street | | scarf-related injuries by electric vehicle wheels | strangulation | Qilu evening papers |
| 113 | 2015/4/25 | Zhejiang | | 4 | boy | | kindergarten | | mental zipper with trousers | genital injured | http://roll.sohu.com/20150804/n418171112.shtml |
| 114 | 2015/2/7 | Heilongjiang | 4 | | boy | kindergarten | | drawstrings | | death | http://www.sohu.com/a/210026468_801371 |
| 115 | 2015/4/9 | Heilongjiang | 7 | | boy | supermarket | | scarf entanglements by electric wheels | | suffocation | https://www.dbw.cn |

**Table S1 Characteristics of included cases(continued)**

| **No** | **Year /month/data** | **Provinces** | **Years** | **Gender** | | **Locations** | **Occurrence reasons** | **Injury outcomes** | **Data sources** |
| --- | --- | --- | --- | --- | --- | --- | --- | --- | --- |
| 116 | 2015/2/9 | Jiling | 5 | boy | | kindergarten | mental zipper with trousers | genital was injured | https:// www .ennews.xwh.cn |
| 117 | 2015/3/1 | Guandong | 4 | boy | | shopping centers | Cocos-shoes elevator | five toes by cut off | https://www.qingdaonews.com |
| 118 | 2015/3/15 | Anhui | 3 | boy | | home | his clothing catch fire | severe burns(45% | https://www.finance.sina.cn |
| 119 | 2011/12/27 | Zhejiang | 10 | girl | | street | scarf-entanglements by vehicle wheels | fatal asphyxiation | https://www.qdrunyang.cn |
| 120 | 2015/3/30 | Heiongjiang | 4 | boy | | kindergarten | the cord of his clothing caught in the slid | fatal asphyxiation | https:**//**www.dwn.cn |
| 121 | 2015/4/1 | Guangdong | <1y | boy | | school | mental zipper with trousers | genital injured | Journal of sicuan medical |
| 122 | 2015/4/15 | Taiwan | <1y | girl | | home | sock threat involved | figure injured | www.huanqiu.com |
| 123 | 2015/4/25 | unknown | 3 | boy | | home | mental zipper with trousers | genital injured | https://www.bbaqw.com/cs/178979.htm |
| 124 | 2015/4/24 | Hubei | 3 | boy | | unstated | mental zipper with trousers | genital was injured | https://www.bbaqw.com/cs/178979.htm |
| 125 | 2015/4/27 | Hubei | 2 | girl | | unstated | button down five pearls from her trousers then push them into his noses | almost suffocation | https:// www ctdsb.cnhubei.com |
| 126 | 2015/5/27 | Guangxi | 6 | girl | hospital | | synthetic skirt catch on fire | burning | https:// www tv.gxtv.cn/tv-4.html |

**Table S1 Characteristics of included cases(continued)**

| **No** | **Year /month/data** | **Provinces** | **Years** | **Gender** | **Locations** | **Occurrence reasons** | **Injury outcomes** | **Data sources** |
| --- | --- | --- | --- | --- | --- | --- | --- | --- |
| 127 | 201/8/4 | Fujian | 10 | boy | school | zipper struck his genital | genital was njured | http://www.fjsen.com |
| 128 | 2015/6/17 | Jiangsu | 25d | boy | home | sock involved | figures were injured | http://www.js.ifeng.com |
| 129 | 2015/7/11 | Jiling | 10 | girl | home | synthetic skirt | burning | http://www.sohu.com/a/22880311 |
| 130 | 2015/8/1 | Beijing | 5 | boy | shopping center | Crocs-shoes elevator | burning | Beijing young daily |
| 131 | 2015/9/20 | Sicuan | 2 | girl | home | ignited a lighter to burn his skirt | severe burn injuries | Chongqing morning daily |
| 132 | 2015/10/15 | Henan | 2 | girl | unstated | push grains into his vaginal | vaginal  infections | Wuhan evenings news |
| 133 | 2015/10/11 | Heibei | 3 | boy | unstated | mental zipper | genital were injured | http:// www news.ifeng.com |
| 134 | 2015/11/1 | Jiangsu | 8 | girl | street | scarf entanglements by electric wheels | spinal fracture | http:// www blog.sina.com.cn/s/blog161.html |
| 135 | 2015/11/16 | Heilongjang | 5 | girl | street | clothing too long entanglements the bus | death | http://www.shzhidao.cn |
| 137 | 2016/1/2 | Zhejiang | 12 | girl | street | scarf entanglements by electric wheels | asphyxiation | hzdaily.hangzhou.com.cn |
| 138 | 2016/1/2 | Niaoning | 4 | girl | home | swallow decorative items with clothing | ear were infected | https://www.dbw.cn |
| 139 | 2016/1/5 | Henan | 8m | boy | home | drawstring with cap | death | https://www.Luoyang evening news |

**Table S1 Characteristics of included cases(continued)**

| **No** | **Year /month/data** | **Provinces** | | **Years** | **Gender** | | **Locations** | | **Occurrence reasons** | **Injury outcomes** | **Data sources** |
| --- | --- | --- | --- | --- | --- | --- | --- | --- | --- | --- | --- |
| 140 | 2016/4/19 | Fujian | | 1 | girl | | swimming poor | | wear open pant resulted infected | infected venereal disease | http://www.360doc.com/content/16/0430/15/10841163_4.shtml |
| 141 | 2016/4/24 | Anhui | | 5 | boy | | children hospital | | swallow decorative items with clothing | his throat were injured | http://www.wanjia hotline |
| 142 | 2016/5/20 | Shandong | | <1y | boy | | home | | glove-involved | two fingers were cut off | http://m.sohu.com/a/2133868 |
| 143 | 2016/7/25 | Anhui | | 10 | girl | | kitchen | | his clothing catch fire when cooking dinner | severe burns | http://www.anhuinews.com |
| 144 | 2016/8/27 | Anhui | | 5 | boy | | unstated | | mental zipper | genital was injured | http://www.sina.com.cn |
| 145 | 2016/8/27 | Shandong | | 3 | boy | | unstated | | mental zipper | genital wasinjured | http://www.chunyuyisheng.com |
| 146 | 2016/9/8 | Hubei | | 3 | girl | | unstated | | clothing catching fire | Experienced serve burns | http://www.cnnb.com.cn |
| 147 | 2016/9/8 | Jiangsu | | 7 | girl | | school | | wear open pant resulted push battles into his vagina | vaginitis | https://m.sohu.com/a/113945942_356072 |
| 148 | 2016/10/27 | Henan | | 2 | boy | | unstated | | mental zipper | genital wasinjured | https://weibo.com/yzwb365 |
| 149 | 2016/10/27 | Zhejiang | 5 | | boy | hospital | | mental zipper | | genital was injured | http://www.Sina.com.cn |
| 150 | 2016/11/1 | Hangzhou | 12 | | girl | hospital | | scarf entanglements | | asphyxiation | Qianjiang evening news |

**Table S1 Characteristics of included cases(continued)**

| **No** | **Year /month/data** | **Provinces** | | **Years** | **Gender** | | **Locations** | **Occurrence reasons** | **Injury outcomes** | **Data sources** |
| --- | --- | --- | --- | --- | --- | --- | --- | --- | --- | --- |
| 151 | 2016/11/9 | Guangdong | | <1y | boy | | hospital | push decorative items into his mouth | suffering pneumonia | https://www.ycwb.com |
| 152 | 2016/11/7 | Jiangsu | | 3 | girl | | sweet | scarf was entanglements by elevator | resulted in asphyxiation | https://www.abbao.cn/paper/1 |
| 153 | 2016/12/1 | Jiangsu | | 5 | boy | |  | swallow the mental zipper into his throat | esophageal be injuries | http://www.sohu.com/a/283608741_100281680 |
| 154 | 2016/12/8 | Shandong | | 2y | boy | |  | open pan his genital was struck by tony car | his genital were injured | www.sdchina.com |
| 155 | 2016/12/18 | Anhui | | 35d | boy | | hospital | socks thread wrapped around his feet | his toes were injured | https://www.mgtv.com/b/327910/4928331.html |
| 156 | 2016/12/18 | Ganshu | | <1y | girl | | home | glove thread involved | toe ischemic necrosis | https://news.ifeng.com |
| 157 | 2016/12/26 | unstated | | 1 | boy | |  | push decorative with his clothing into his mouth | pneumonia | https://www.ycwb.com |
| 158 | 2016/12/31 | Hubei | | <1y | boy | |  | gloves involved | Eye were injured | https://www.douban.com/group/topic/95599215/ |
| 159 | 2017/2/6 | Hubei | | 5 | girl | |  | push coins into her vaginal | bleeding, infections | people.rednet.cn/people .daily |
| 160 | 2017/2/28 | Zhejiang | | 2 | boy | |  | glove thread involved | face bleeding | https://www.mgtv.nb.zjol.com.cn/sh |
| 161 | 2017/3/1 | Shanxi | 4 | | boy |  | | mental zipper-related | genital was injured | https://www.mgtv.sjpd.zynews.cn/ |
| 162 | 2017/3/2 | Sicuan | 2 | | girl |  | | clothing catch fire | experience burns | https://www.sicuan news |

**Table S1 Characteristics of included cases(continued)**

| **No** | **Year /month/data** | **Provinces** | | **Years** | **Gender** | | **Locations** | **Occurrence reasons** | **Injury outcomes** | **Data sources** |
| --- | --- | --- | --- | --- | --- | --- | --- | --- | --- | --- |
| 163 | 2017/3/27 | Guangxi | | <1y | girl | |  | swallow decorative with his clothing | esophagus injuries | https://www.familydoctor.cn/a/201703/1787874.html |
| 164 | 2017/3/30 | Henan | | 5 | girl | | hospital | push coins into her vigor | vaginal bleeding | Zhengzhou evening news |
| 165 | 2017/4/12 | Zhejiang | | 5 | boy | | school | figure was cut off because tie with his clothing | Figures were injured | Wenzhou evening news |
| 166 | 2017/4/22 | Shanghai | | <1y | boy | | hospital | swallow decorative items with clothing | suffocation | zqb.cyol.com ,www.cyol.com |
| 167 | 2017/4/22 | Hubei | | 2 | girl | | home | push grains into his vaginal | vaginal bleeding | http://www.sohu.com/a/30972512399903362 |
| 168 | 2017/4/24 | Fujian | | <1y | girl | | hospital | open seat pants | fall in the elevator | https:// https://www.baike.baidu.com/ |
| 169 | 2017/4/29 | Jiangxi | | <1 | boy | |  | swallow decorative items with his maps | head was injured | https://www.cssqt.com |
| 170 | 2017/5/5 | Anhui | | 4 | girl | | hospital | push clothing’s marbles into her vagina | urinary tract infection | https://[www.zjol.com.cn/](http://www.baidu.com/link?url=LNrnLdhiXTnPNM2zp-vWJ9TLl0NHVPi2bRuE0UA1pXeTvae1dlPUhqk9kV8IPfSe) |
| 171 | 2017/5/4 | Jiangsu | | 3 | girl | | unstated | wear open pan push battles into his vagina | vaginitis | https://www.jhcb.net/index.php- |
| 172 | 2017/5/8 | Henan | 3 | | girl | unstated | | mental zipper | eyelid was injured | Yangtse evening news |
| 173 | 2017/5/9 | Sichuan | 2 | | boy | unstated | | wear open pan push battle into his vagina | his genital injured by animal bites | https://www.wuhunews.cn/ |

**Table S1 Characteristics of included cases(continued)**

| **No** | **Year /month/data** | **Provinces** | | **Years** | **Gender** | | **Locations** | | **Occurrence reasons** | **Injury outcomes** | **Data sources** |
| --- | --- | --- | --- | --- | --- | --- | --- | --- | --- | --- | --- |
| 174 | 2017/5/15 | Shandong | | 2 | boy | | street | | wear open pan playing “yaokong” car | his genital was injured | http://www.sohu.com/a/156913179_526721 |
| 175 | 2017/5/24 | Hubei | | 1 | boy | | hospital | | wear open pan and falling in the elevator | his genital was injured | https://mini.eastday.com/a/180825.html |
| 176 | 2017/6/1 | Guangdong | | <1y | girl | | hospital | | a turtleneck sweater | neck alleged | https:// www xywy.com |
| 177 | 2017/6/3 | Unstated | | 3 | girl | | unstated | | swallowed decorative items with clothing | throat injured | https://www .newpaper.dahe.cn |
| 178 | 2017/6/10 | Anhui | | 7 | girl | | home | | his skirt catch fire | burning | https:/ www /ah.ifeng.com/ |
| 179 | 20176/12 | Guangdong | | 27days | boy | | streets | | wear open pan | his genital was injured by dog | http://m.sohu.com/a/148337308_809076 |
| 180 | 2017/6/23 | Fujian | | 5 | girl | | kindergarten | | anit-sunny clothing | skin allegic reactions | https://www ://szb.qzwb.com/dnzb/html.. |
| 181 | 2017/7/13 | Shandong | | 4 | girl | | shopping centers | | Crocs-shoes | his two toes was injured | http://www.sohu.com/a/156925480_799858 |
| 182 | 2017/7/15 | Shanxi | 1 | | boy | community | | open seat pants | | burning | http://www.szb.qzwb.com/dnzb/html. |
| 183 | 2017/7/26 | Jiangsu | 4 | | boy | unstated | | zipper with trousers | | his genital was injured | https://www.39.net |
| 184 | 2017/8/16 | Fujian | 5 | | boy | unstated | | zipper with trousers | | his was injured | https://www.yihu.com/doctorArticle.shtml |
| 185 | 2017/10/24 | Hubei | 1 | | boy | unstated | | zipper with trousers | | his was injured | https://www sina.com.cn |

**Table S1 Characteristics of included cases(continued)**

| **No** | **Year /month/data** | **provinces** | **Years** | **Gender** | **Locations** | **Occurrence reasons** | **Injury outcomes** | **Data sources** |
| --- | --- | --- | --- | --- | --- | --- | --- | --- |
| 186 | 2017/11/22 | Hubei | 6 | boy | school | drawstring with his jackets | suffocation | http://www.sohu.com/a/205946270_116316 |
| 187 | 2017/12/1 | Guangxi | 5 | boy | kindergarten | mental zipper with trousers | his genital wasinjured | [www.nntv.cn/live/nntv_...](https://www.baidu.com/link?url=TFkcYlK8vtExuVQU8ZrI8qu3HR_uQ0bKXyYCdqh6Os6OJAczo2SIenVs0rl6WfvZ&wd=&eqid=be12a7f9000af4e6000000065db56c62) |
| 188 | 2017/12/9 | Hunan | 9 | girl | school | mental zipper struck with his coat | his eye was injured | https://www.chunyuyisheng.com/pc/article/133599/ |
| 189 | 2017/12/11 | Fujian | 3 | girl | home | synthetic skirt catching fire | experience severe burns | https://tv.sohu.com/user/33119 |
| 190 | 2017/12/25 | Hubei | 1 | boy | supermarket | open seat pants | genital was injured | https://www.hbrb.cnhubei.com/ |
| 191 | 2017/12/27 | Guangdong | 3 | boy | hospital | swallowed the decorative buttons on his clothes for 20 hours | suffocation | https://www.hzdaily.hangzhou.com.cn/ |
| 192 | 2017/12/29 | Hunan | <1y | boy | home | wrap baby toes in a sock thread for 6 days | four toes were severely injured | https:// www.Xiaoxiang Morning News |
